# A Revised Probabilistic Estimate of the Maternal Methyl Mercury Intake Dose Corresponding to a Measured Cord Blood Mercury Concentration

**DOI:** 10.1289/ehp.7417

**Published:** 2004-11-04

**Authors:** Alan H. Stern

**Affiliations:** Division of Science Research and Technology, New Jersey Department of Environmental Protection, Trenton, New Jersey, USA; and Division of Environmental and Occupational Health, University of Medicine and Dentistry of New Jersey–School of Public Health, Piscataway, New Jersey, USA

**Keywords:** cord blood, maternal, mercury, methyl mercury, Monte Carlo, one-compartment, pharmacokinetic, probabilistic, reference dose, RfD

## Abstract

In 2001, the U.S. Environmental Protection Agency (EPA) adopted a revised reference dose (RfD) for methyl mercury (MeHg) of 0.1 μg/kg/day. The RfD is based on neurologic developmental effects measured in children associated with exposure *in utero* to MeHg from the maternal diet. The RfD derivation proceeded from a point of departure based on measured concentration of mercury in fetal cord blood (micrograms per liter). The RfD, however, is a maternal dose (micrograms per kilogram per day). Reconstruction of the maternal dose corresponding to this cord blood concentration, including the variability around this estimate, is a critical step in the RfD derivation. The dose reconstruction employed by the U.S. EPA using the one-compartment pharmacokinetic model contains two areas of significant uncertainty: It does not directly account for the influence of the ratio of cord blood:maternal blood Hg concentration, and it does not resolve uncertainty regarding the most appropriate central tendency estimates for pregnancy and third-trimester–specific model parameters. A probabilistic reassessment of this dose reconstruction was undertaken to address these areas of uncertainty and generally to reconsider the specification of model input parameters. On the basis of a thorough review of the literature and recalculation of the one-compartment model including sensitivity analyses, I estimated that the 95th and 99th percentiles (i.e., the lower 5th and 1st percentiles) of the maternal intake dose corresponding to a fetal cord blood Hg concentration of 58 μg/L are 0.3 and 0.2 μg/kg/day, respectively. For the 99th percentile, this is half the value previously estimated by the U.S. EPA.

In 2001, the U.S. Environmental Protection Agency (EPA) adopted a revised reference dose (RfD) for methyl mercury (MeHg) of 0.1 μg/kg/day (U.S. EPA 2001a, 2001b; Rice et al. 2003), relying heavily on the assessment conducted by the National Research Council (NRC 2000). The RfD is based on neurologic developmental effects measured in children associated with exposure *in utero* to MeHg from the maternal diet. The NRC and U.S. EPA assessments employed a benchmark dose approach to derive the lower 95% confidence interval on the fetal cord blood mercury concentration, doubling the proportion of children in the lowest 5% of performance on tests of neurologic performance. The NRC identified a cord blood concentration of 58 μg/L (ppb) total Hg based on analysis of the individual test judged to give the most sensitive and robust response, whereas the U.S. EPA identified a range of cord blood concentrations of 46–79 μg/L based on consideration of several tests. These values are in fact concentrations, whereas the RfD is a dose—in this case, the maternal intake dose. The reconstruction of the maternal MeHg intake dose that resulted in the observed cord blood Hg concentration is a critical step in the RfD derivation. The dose reconstruction requires a pharmacokinetic model linking dose and blood concentration. Two different types of pharmacokinetic models have been used for this purpose—a physiologically based pharmacokinetic (PBPK) model (Clewell et al. 1999) and the one-compartment model (Stern 1997; Swartout and Rice 2000). In both types of models, the relationship between cord blood concentration and dose depends on several physiologic and metabolic parameters. The values of these parameters vary among individuals. The population variability in the value of these parameters results in variability in the output of these models. Thus, there is no unique relationship between a given cord blood Hg concentration and a single maternal intake dose. Rather, this relationship is described by a probability distribution. For the RfD to be appropriately protective and inclusive of the variability in the population, the estimate of intake dose must itself be a distribution that describes this variability. This requires that the inputs to these pharmacokinetic models be in the form of probability distributions. The calculation of the outputs of these models using probability distributions has been accomplished through the use of Monte Carlo simulation. Both the PBPK and pharmacokinetic models for MeHg have been analyzed in this manner and have yielded estimates of variability in maternal dose reconstruction that are quite comparable. These include two separate analyses of the one-compartment model (Stern 1997; Swartout and Rice 2000) and one analysis of the PBPK model (Clewell et al. 1999; Stern et al. 2002).

Despite the close agreement regarding variability among these analyses, significant uncertainty remains regarding the appropriate central tendency estimates (e.g., means, medians) for the model parameters and, consequently, for the output of the model. This uncertainty results from the differing assumptions and different data employed among these three analyses (NRC 2000). In part, these differences result from uncertainty as to whether these parameters need to reflect conditions during pregnancy and from lack of specificity as to the period of pregnancy. Recognizing this source of uncertainty, the NRC (2000) assessment recommended separating the central tendency and variability aspects of the dose reconstruction. This approach was adopted by the U.S. EPA (2001b). In this approach, nondistributed (i.e., single value) central tendency estimates were selected for each parameter of the one-compartment model. The resulting output of the model represented a central tendency estimate of the maternal intake dose. This value was divided by an uncertainty factor derived from the analysis of variability generated from the probabilistic analysis of the distributions of the parameters of the one-compartment model. The value of the uncertainty factor was normalized to the central tendency estimate based on the ratio of the 50th percentile to the 1st percentile (i.e., lower 99th percentile) of the distribution of the dose derived from variability analysis. The resulting value is an estimate of the dose that accounts for 99% of the interindividual variability in the dose reconstruction (NRC 2000; Stern et al. 2002). Although this approach makes the uncertainty in the selection of the central tendency estimates explicit, it does not resolve that uncertainty.

Another significant source of uncertainty in the dose reconstruction was the failure to adequately incorporate the cord blood:maternal blood Hg ratio. The one-compartment model specifically estimates the relationship between maternal MeHg intake and maternal blood Hg concentration. To extend this relationship to fetal cord blood Hg concentration, it is necessary to estimate the maternal blood concentration corresponding to a measured cord blood concentration. This is done by applying an empirically derived ratio. In the NRC (2000) assessment, evaluation of a limited set of data led to the suggestion that this ratio was close to 1.0. On the basis of that conclusion, the benchmark cord blood Hg concentration was assumed to adequately describe the corresponding maternal blood Hg concentration. A preliminary analysis of the literature by the U.S. EPA (2001) suggested that the correct value for this ratio was likely to be > 1.0. The U.S. EPA RfD derivation thus included the uncertainty in the cord blood:maternal blood Hg ratio as a factor in its overall uncertainty factor adjustment. Nonetheless, the U.S. EPA did not identify a central tendency estimate for the cord:maternal ratio and did not account for the variability around that estimate. Thus, the appropriate contribution of this ratio to the overall RfD remains an unresolved source of uncertainty.

In an attempt to resolve both of these sources of uncertainty and to examine the extent to which a more complete analysis might alter the value currently employed in the RfD derivation, I have undertaken a reanalysis of the dose reconstruction. I have revisited the scientific literature and have reevaluated both central tendency estimates and overall distributions for each of the input parameters in the one-compartment model with a specific emphasis on pregnancy and, more specifically, on third-trimester–specific values. I have also incorporated the results of a recent analysis of the distribution of the cord blood:maternal blood Hg ratio (Stern and Smith 2003) into the overall analysis of the maternal dose reconstruction.

## The One-Compartment Model

For consistency with the existing U.S. EPA RfD approach for MeHg, in this analysis I focus on the one-compartment model rather than the PBPK model for dose reconstruction. The one-compartment model is used in contrast to the PBPK model because it employs a relatively small set of parameters whose distributions within the population are generally well characterized. This facilitates a more accurate and transparent assessment of variability in the dose estimate. The one-compartment model predicts the relationship between MeHg intake dose and MeHg blood concentration under steady-state conditions in a closed system—in this case, the maternal system. The model is expanded to address the relationship between maternal intake dose and fetal cord blood by applying an empirically derived ratio to estimate the maternal blood concentration corresponding to the measured cord blood concentration.

The one-compartment model [NRC 2000; World Health Organization (WHO) 1990] as modified to include the cord blood:maternal blood Hg ratio, can be expressed as


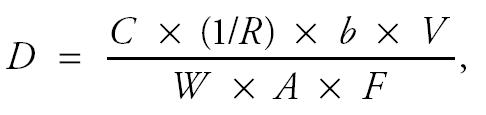


where *D* is the maternal intake dose of MeHg (micrograms per kilogram per day), *C* is the measured Hg concentration in cord blood (for this analysis, the cord blood concentration is assumed to be the BMD (benchmark dose) value of 58 μg/L identified by the NRC (2000), *R* is the ratio of cord blood Hg concentration/maternal blood Hg concentration (unitless), *b* is the rate constant for elimination of MeHg from the blood (per day), *V* is the maternal blood volume (liters), *W* is the maternal body weight (kilograms), *A* is the fraction of the ingested dose that is absorbed (unitless), and *F* is the fraction of the absorbed dose that is present in the blood at steady state (unitless).

## Materials and Methods

I searched the scientific and medical literature for data relating to the parameters of the one-compartment model. Priority was given to data for pregnant women, particularly data specific to the third trimester of pregnancy. To generate both central tendency estimates and descriptions of the distributions of the parameters, data were selected only if they were available as subject-specific values, summary percentiles, or graphic representations from which distributional data could be deduced (e.g., histograms). When two different appropriate data sets were available for a model parameter, the determination of whether to combine the data sets was based on a test (*t*-test and/or the nonparametric Mann-Whitney test as appropriate) of the hypothesis that the two data sets arose from the same underlying distribution. Data were tested for fit to parametric distributions (generally normal or log-normal) using curve-fitting software (BestFit for Windows, version 2.0d; Palisade Corp., Newfield, NY). In the absence of reasonable fits to parametric distributions, distributions were described by empirical distributions (e.g., cumulative probability distributions). Probabilistic (Monte Carlo) analysis of the one-compartment model was carried out using @Risk for Windows (version 3.5.2; Palisade Corp.). Latin hypercube sampling was employed to provide adequate representation of the tails of distributions. Sampling was accomplished with 5,000 iterations because this gave good interiteration stability in the moments of the output distribution. Results of the model simulations are based on the average of five separate simulations of 5,000 iterations each. Sensitivity analyses of central tendency estimates and variability results were carried out as indicated in the text. Unless otherwise indicated, all statistical analyses were carried out using Statistica (version 6.1; StatSoft Inc., Tulsa, OK).

## Derivation of Probability Distributions for Model Parameters

Table 1 summarizes the probability distributions derived for each of the parameters in the one-compartment model. The detailed rationale for each parameter follows. In each case, an estimate of the true uncertainty (i.e., lack of knowledge) in the derivation of each parameter is provided. Consistent with the lack of knowledge, this estimate is semiquantitative using a relative scale of high, medium, and low uncertainty, based on professional judgment. This estimate is then employed in the subsequent sensitivity analysis.

### C—*the measured concentration of Hg in fetal cord blood.*

This is an empirically derived constant. It is a biomarker of fetal MeHg exposure derived from the benchmark dose analysis relating neurodevelopmental performance to the measured concentration of total Hg in cord blood (NRC 2000). Although the benchmark dose analysis is subject to uncertainty, this value, once derived, is carried forward as the point of departure in the pharmacokinetic analysis. The uncertainty in this value is dealt with elsewhere in the overall risk assessment. The NRC (2000) identified a value of 58 μg/L based on its selection of the most appropriate test of developmental performance from the Faroe Islands study. The U.S. EPA (2001a, 2001b) identified a range of values bracketing the NRC selection from several tests in that study. To simplify this analysis, the single value of 58 μg/L is used. Because this value is a constant, the variability in the dose estimate is unaffected by the specific value selected, and the central tendency estimate of the dose scales linearly with the selected value. This value reflects measurement of the concentration of total Hg. As discussed below, and in detail by Stern and Smith (2003), the correct value for the purposes of dose reconstruction is the concentration of MeHg + MeHg-derived inorganic Hg. This value is closely estimated by the concentration of total Hg in cord blood.

### R—*the cord blood:maternal blood Hg ratio.*

The derivation of this value is discussed in detail by Stern and Smith (2003). Briefly, the ratio was calculated from 10 separate studies meeting the selection criteria. The mean and standard deviation of the ratio from each study were calculated from the raw data, or estimated by probabilistic simulation, and showed a generally close agreement across studies for both parameters. Each study was described as the corresponding log-normal distribution, and the distributions from the individual studies were sampled in a meta-analysis to generate a summary distribution. The recommended summary distribution, mean ± SD = 1.7 ± 0.93, is used in this analysis. This ratio has historically been expressed as cord blood Hg/maternal blood Hg. However, the one-compartment model requires the reciprocal form. To avoid confusion, the historical form of the ratio is retained, and its reciprocal (1/*R*) is calculated in the model.

Based on the analysis by Stern and Smith (2003), an estimate of low true uncertainty is applied to this parameter.

### b—*the elimination rate constant for MeHg in maternal blood.*

As noted above for *C*, total Hg rather than MeHg was measured in cord blood. Smith et al. (1994) and Smith and Farris (1996) point out that if the half-life in the blood of a dose of MeHg is measured as MeHg per se, it will be shorter than if it is measured as total Hg. This occurs because, even though the inorganic metabolite is not rapidly cleared from the blood, it is no longer measured as MeHg. The authors therefore suggest expressing the elimination rate of MeHg from the blood based on MeHg-specific measurements. This is appropriate if the rate of MeHg elimination is employed to directly estimate the period of time during which MeHg is present in the blood to exert a toxic effect (i.e., area-under-the-curve considerations). In this analysis, however, the elimination rate of MeHg is employed to calculate the steady-state balance between the ingestion of MeHg and its elimination. For this purpose, the inherent toxicity of MeHg does not enter into consideration. Thus, the appropriate metric is the rate of elimination of all the Hg that entered the blood as ingested MeHg. In maternal blood, this is closely estimated by the concentration of total Hg (NRC 2000; Stern and Smith 2003).

Three studies report half-lives/elimination rates measured directly in blood from nonpregnant females as well as males, Sherlock et al. (1984), Miettinen et al. (1971), and Kershaw et al. (1980). In the study by Miettinen et al. (1971), five men and one woman ate a single fish meal containing added ^203^Hg. The total mass of Hg in the fish meal, including endogenous Hg, was 22 μg. Neither the dose nor the body weight of the subjects is presented. However, if a body weight of 70 kg is assumed, the dose can be estimated at 0.3 μg/kg. The mean half-life was 49.87 days. Kershaw et al. (1980) administered an intravenous dose of MeHg ranging from 18.1 to 21.8 μg/kg/day to four men. The mean half-life was 52.98 days. Al-Shahristani and Shihab (1974) measured the decrease in Hg concentration along hair strands of 48 subjects of both sexes and ages from 6 months to 66 years in Iraq who were defined as “patients” as a result of the MeHg poisoning epidemic 8–12 months previously. The mean half-life was estimated to be 72 days with a wide range (36–189 days). The maximum hair concentrations are not provided, and the corresponding intake doses cannot be estimated from the available data. However, given that the subjects were still recognized as patients up to a year after the exposure, it is likely that their exposures were quite high. Because of the likelihood that the exposures resulted in frank toxicity, and given the inability to address the extent to which these elevated exposures may have affected the half-life (see below), the data from Al-Shahristani and Shihab (1974) were not further considered.

Cox et al. (1989) presented data from the Iraqi poisoning episode for 55 women (of the initial cohort of 83) who were exposed to MeHg-treated grain while pregnant and whose exposure was sufficiently large to allow x-ray fluorescence analysis of Hg in single hair strands. The decrease in Hg concentration was followed in their hair after they eliminated the MeHg-treated grain from their diets. Data on individual elimination rate as a function of exposure are not provided, but it is reported that hair concentration ranged from 10 to 670 ppm. This range would correspond to a steady-state daily intake of approximately 1.2–79.6 μg/kg/day assuming a 62-kg woman (NRC 2000). The population mean dose is not given. Data were presented for 122 hair samples from the 55 subjects. For generating summary and distributional statistics, it was assumed that each subject contributed equally to the total number of analyzed hair strands. Data were presented by Cox et al. (1989) as a histogram with the half-life binned in 5-day intervals. The frequency for the midpoint value for each half-life bin was read off the graph. The mean half-life for the 122 samples was 47.17 days. Although these data do not specifically represent third-trimester conditions, they are the only elimination rate data reported for a pregnant population and therefore appear to be the most applicable to the present analysis. However, the elevated level of exposure in this population raises concerns that the elimination rate may vary as a function of dose. Sherlock et al. (1984) present data on half-life and dose for 20 subjects given an experimental dose of MeHg. The subjects ingested MeHg in prepared fish meals two to four times per week over a 96-day period. The mean half-life was 50.20 days. The doses varied within a relatively small range from 0.5 to 3.6 μg/kg/day (mean = 1.6 μg/kg/day). The mean dose in the Sherlock et al. study is encompassed by the estimated dose range among the subjects in the Cox et al. (1989) study. It is clear, however, from the Cox et al. report that a significant fraction of the study population had exposures in the upper end of the range. In the Sherlock et al. (1984) data set, the half-life is significantly correlated with the dose (*r*_Spearman_ = 0.52). Based on linear regression, the half-life increased by 3.21 days for each microgram per kilogram per day increase in dose (*p* = 0.04). The nominal half-life at zero dose (i.e., the y-intercept) is 45 days.

As shown in Table 2, with the exception of the Cox et al. (1989) data, the mean half-lives of the studies for which the dose is known or can be derived increase with increasing MeHg dose. This is consistent with the relationship observed in the Sherlock et al. (1984) data. Thus, although the upper range of exposures in Cox et al. (1989) exceeds the doses in the other studies by a factor of at least four, that study yields the smallest half-life, with a mean value close to that predicted at zero dose from the Sherlock et al. (1984) data. Note, however, that the Cox et al. (1989) estimate is specific to the period of pregnancy. During pregnancy, MeHg crosses the placenta and is retained in the fetal compartment. Given a mean cord blood:maternal blood ratio of 1.7 (Stern and Smith 2003), the transfer of MeHg to the fetus is, in essence, an additional route of maternal elimination and would be expected to result in a shorter half-life in pregnant women than in nonpregnant adults. Nonetheless, maternal elimination of MeHg across the placenta does not preclude a concentration dependence for other routes of maternal elimination. There does not appear to be any reliable way to account for the relative influence of each of these competing processes and thus to quantitatively adjust the half-life estimate from Cox et al. (1989) to account for concentration dependence. The Cox et al. data are therefore selected as the most appropriate estimate of half-life (and the elimination rate constant) during pregnancy with the caveat that the true value of the central tendency for lower doses may be smaller than the reported value. It should, however, be noted that, given the relatively wide range of exposures represented in this data set, the estimate of variability may be less uncertain than the central tendency estimate.

**Figure 1:**
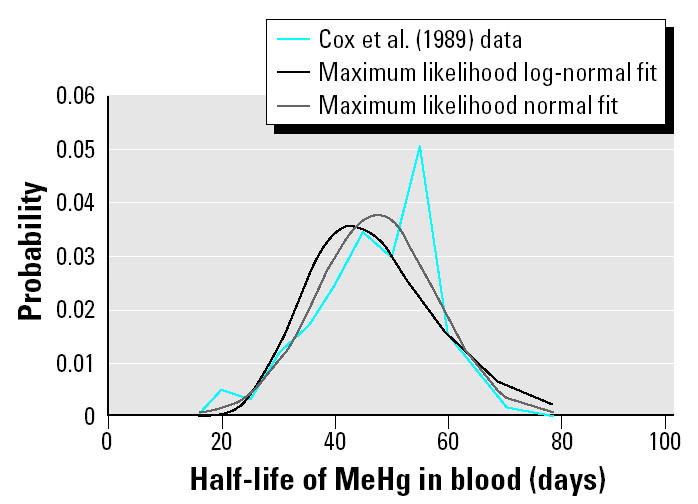
Comparison of Cox et al. (1989) half-life data to maximum likelihood normal and log-normal distributions.

As shown in Figure 1, these data are not closely fit by parametric distributions, and there is a suggestion of a bimodal distribution. They are therefore described by an empirical distribution whose minimum and maximum are calculated as mean ± 3 SD.

The pregnancy-specific nature of the Cox et al. (1989) half-life data, and its lower mean value, consistent with fetal transport, implies that the data are relatively specific. The methodology of tracking elimination through hair segments is also relatively accurate. On the other hand, the possibility that the central tendency estimate is biased high due to the high dose to this population produces some uncertainty in this estimate. An overall estimate of medium true uncertainty is therefore assigned to this parameter.

### V—*the maternal blood volume.*

Given the half-life of MeHg in maternal blood, the concentration in cord blood reflects maternal exposures during the last half of the third trimester (NRC 2000). Therefore, the most appropriate measure of maternal blood volume (*V*) is the blood volume during the third trimester of pregnancy. Blood volume increases during pregnancy starting at the sixth to eighth week and reaching a maximum at 32–34 weeks with a volume about 40% larger than the nonpregnant state (Fredriksen 2001).

Limited data on third-trimester blood volumes in the United States from which estimates of population variability can be derived are available from early work. Only data generated < 30 days before delivery were considered for the purposes of this analysis. Thomson et al. (1938) present data on third-trimester blood volume for 11 women (mean ± SD = 5.42 ± 0.92 L). Caton et al. (1951) present third-trimester data for 10 women (mean ± SD = 5.74 ± 0.97 L). Analysis of these data sets indicates that they are consistent with the hypothesis that both arise from the same underlying distribution (*p_t_*_-test_ = 0.44). They are therefore combined into a single data set (*n* = 21, mean ± SD = 5.57 ± 0.93 L). Although these data are relatively old, Hytten (1985), in summarizing the more recent state of knowledge of blood volume at 40 weeks of pregnancy, reported a value of 5.50 L. The earlier studies thus appear to compare well with more recent data. Thomson et al. (1938) also report the measured body weight corresponding to the blood volumes. Third-trimester blood volume and body weight are moderately correlated (*r*_Spearman_ = 0.49), but the correlation is marginally significant (*p* = 0.13). Body weight and blood volume are known to be correlated in general (Fredriksen 2001; Hytten 1985). Therefore, the lack of significance for this correlation in the Thomson et al. (1938) data likely reflects the small sample size rather than the absence of a meaningful correlation.

**Figure 2:**
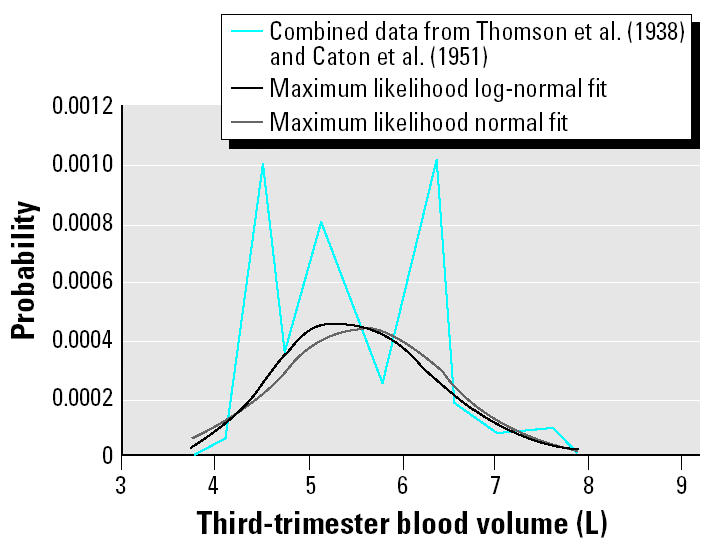
Combined third-trimester blood volume data from Thomson et al. (1938) and Caton et al. (1951) compared with maximum likelihood normal and log-normal distributions.

As seen in Figure 2, the combined data set is not well described by parametric distributions, and there is a suggestion of polymodality. These data are therefore described as an empirical distribution, with minimum and maximum values chosen as mean – 2 SD and mean + 2.5 SD, respectively. The nonsymmetrical bounds are chosen to accommodate the right-skewness of the data. In the probabilistic analysis, samples of *V* and maternal body weight at delivery (*W*) are mutually constrained by the correlation coefficient derived from the Thomson et al. (1938) data.

These data are highly specific to the target population. Although the combined data set for this parameter is relatively small, the overall variance is also small (coefficient of variation = 0.17), as would be expected given the physiologic constraints on blood volume. Thus, it is not likely that a larger sample would significantly change the parameters of the resulting distribution. Despite the age of the data, they appear consistent with more contemporary estimates. This parameter is therefore judged to have a low true uncertainty.

### A—*the fraction of the dose that is absorbed.*

This parameter is a measure of the fraction of the mass of ingested MeHg that is absorbed into the body as a whole. As such, it cannot be measured directly by sampling of blood but requires either whole-body counting of labeled MeHg or long-term monitoring of Hg^2+^ and MeHg excretion in the absence of prior or subsequent confounding exposures. Consequently, there are few human data for this parameter. Miettinen et al. (1971) fed measured portions of fish containing a known activity of radio-labeled MeHg to 14 adult subjects, six females and eight males. Whole-body counting was used to measure the amount of activity remaining after 3–4 days, and urine and feces counting was used to measure the excreted activity during that period. Miettinen et al. (1971) used these measurements to estimate the fraction of the intake dose that was retained/absorbed. Measurements were taken after the 3–4 day period presumably to allow elimination of MeHg that had become associated with the lining of the gastrointestinal tract but not absorbed into the body proper. The mean retention was estimated at 94%. However, during this 3–4 day period, some fraction of the absorbed dose was metabolized to Hg^2+^ and excreted. The original Miettinen et al. (1971) estimate does not account for this metabolism and thus slightly underestimates the correct value of this parameter. Previous assessments of the one-compartment model (Stern 1997; Swartout and Rice 2000) used this original Miettinen et al. estimate. However, Miettinen et al. (1971) also provide individual estimates of the whole-body half-life of MeHg. This allows an estimate of the fraction of the absorbed activity that was eliminated during the 3–4 days due to metabolism. The corrected value of *A* is estimated as *A* = *D*/(*M* + *U*), where *D* is the administered MeHg activity, *M* is the administered MeHg lost to metabolism, and *U* is the administered activity that is retained at 3–4 days.

The value of *A* estimated using this approach (mean ± SD = 0.97 ± 0.016) is 3.4% larger than the original estimate. The mean values for males and females are almost identical, and the entire data set is therefore used to estimate the distribution of *A*. Aberg et al. (1969) also reported that “almost 100%” of administered MeHg label was absorbed. Although the available data do not relate to pregnancy, given the absorption of close to 100% in the nonpregnant state, it does not seem likely that absorption during pregnancy would be substantially altered.

**Figure 3:**
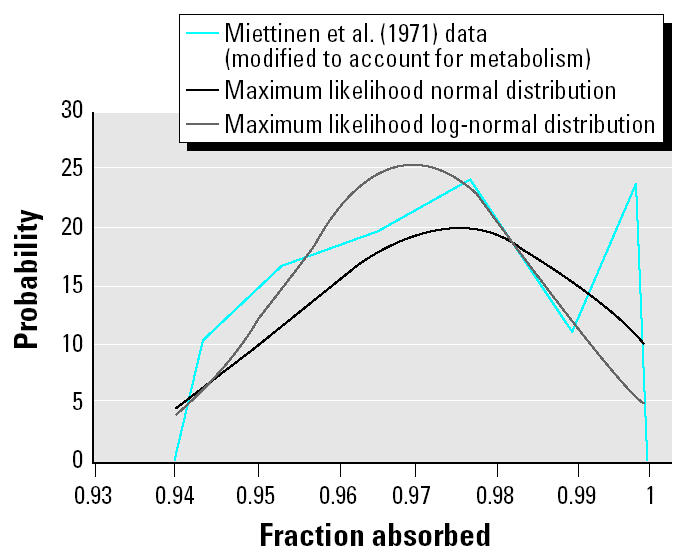
Comparison of Miettinen et al. (1971) data on the fraction of MeHg absorbed to maximum likelihood normal and log-normal distributions.

The data set is not well fitted by any parametric function, and there is some suggestion of a bimodality, with a secondary peak at > 99% absorption (Figure 3). The data are therefore fit to an empirical cumulative probability distribution. Given the narrow range of the data, the minimum and maximum values are selected empirically (0.940 and 0.999, respectively).

Although the available data are not third-trimester– or pregnancy-specific, they are precise and describe a small range of variability. Given the likelihood that these values, apparently resulting from simple uptake phenomena, would not change as a result of the pregnant state, this parameter is judged to have low true uncertainty.

### F—*the fraction of the absorbed dose that is present in the blood at steady state.*

This parameter refers to the fraction of the absorbed dose of MeHg found in the maternal blood after distribution among the various tissues. Functionally, it is derived from the same data as the elimination rate constant by back extrapolating the linear fit of the relationship between time (*T*) and the log of the Hg concentration (or radio-tracer activity) in the blood to *T* = 0. This gives the concentration (or activity) in the blood at the theoretical point at which MeHg in blood had equilibrated with other tissues, but before it started to be eliminated from the blood. This value, however, must be converted to the mass of MeHg in the total blood volume and then expressed as a fraction of the absorbed mass of MeHg. Thus, *F* is calculated from


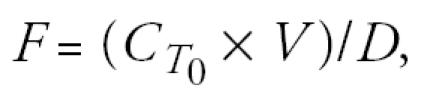


where *C_T_*_0_ is the concentration of MeHg in blood at *T* = 0 (micrograms per liter), *V* is the blood volume (liters), and *D* is the absorbed dose (micrograms).

Sherlock et al. (1984), Kershaw et al. (1980), and Smith et al. (1994) do not report blood volume. Although Kershaw et al. (1980) and Smith et al. (1994) report values for *F*, it appears that blood volumes used in their calculations were based on an assumed deterministic relationship between body weight on blood volume (Allen PV, personal communication). In the case of Kershaw et al. (1980), it appears that blood volume was calculated as 7% of body mass in kilograms. Individual values of *F* for the nonpregnant state can be estimated from Sherlock et al. (1984) based on their data for the percentage of intake dose distributed in 1 kg of blood by assuming a deterministic relationship between body weight and blood volume. Thus, for consistency with the approach in Kershaw et al. (1980), blood volume was estimated for the Sherlock et al. (1984) data by assuming that blood volume (L) is equal to 7% of body weight (kilograms). However, estimation of *F* using such an approach underestimates the overall variability in *F*. For the Sherlock et al. (1984) data, a further deterministic assumption must be made to estimate the volume corresponding to 1 kg of blood (i.e., blood density). This introduces an additional small loss in variability in the distribution of *F*. Standard values for blood density are generally given as 1.06 kg/L (Reinking 2002; U.S. EPA 2002). Miettinen et al. (1971), although providing activity versus time data, provided no data on blood volume or body weight. These data cannot therefore be used to estimate *F* without an arbitrary assumption of blood volume.

The Sherlock et al. (1984) data show a moderately negative correlation between the fraction of the dose in 1 kg of blood and body weight (*r* = –0.47, *p* = 0.04). The authors suggest that this is a function of the larger volume of distribution to be expected with larger body masses. Swartout and Rice (2000) included this correlation in their simulation of the one-compartment model. This explanation is consistent with the positive correlation between body weight (*W*) and blood volume (*V*) of approximately the same magnitude derived from the data of Thomson et al. (1938). However, note that the relationship with body weight derived from Sherlock et al. (1984) refers to the percentage of the intake found in 1 kg of blood. As body weight increases, the total blood volume also increases, and the fraction of the total blood Hg that is found in a fixed volume (or mass) of blood (e.g., 1 kg) decreases proportionally. This will be the case even if the fraction of the MeHg intake found in the total blood volume remains constant. Therefore, the correlation observed by Sherlock et al. (1984) does not appear to reflect a relationship that is independent of the correlation between body weigh and blood volume. Thus, having already specified that correlation in the model inputs for *W* and *V*, it does not appear necessary to additionally specify a correlation between *W* and *F*.

In both Kershaw et al. (1980) and Smith et al. (1994), all subjects were men. In the Sherlock et al. (1984) study, 6 of the 20 subjects were women. The calculated values of *F* for the women (mean = 4.59%) were significantly less than those for the men (mean = 5.58%; *p_t_*_-test_ = 0.014; *p*_Mann-Whitney_ = 0.011). However, multiple regression analysis of *F* shows that when both sex and body weight are included as independent variables, weight is significant (*p* = 0.03) but sex is not. Thus, sex does not appear to have an influence on *F* independent of body weight. The values of *F* calculated from Kershaw et al. (1980; mean = 5.63%) and Sherlock et al. (1984) (mean = 5.28%) are clearly different from those from Smith et al. (1994) (mean = 7.7%; *p*_Kruskal-Wallis_ = 0.005). The Sherlock et al. (1984) and Kershaw et al. (1980) data, on the other hand, are not statistically different (*p*_Mann-Whitney_ = 0.35), the small size of the Kershaw et al. (1980) data set notwithstanding. Therefore, although the Sherlock et al. (1984) and Kershaw et al. (1980) data sets for *F* appear compatible, the Smith et al. (1994) data are incompatible. Differences in body weight between the Smith et al. subjects and those of Sherlock et al. or Kershaw et al. do not appear to account for this difference, and the reason for this difference is not clear. However, it is possible that the unknown relationship employed by Smith et al. (1994) to estimate blood volume from body weight may contribute to this difference. In light of the good agreement between the Sherlock et al. (1984) and Kershaw et al. (1980) estimates of *F*, these data sets are selected as the basis for a distribution for this parameter.

**Figure 4:**
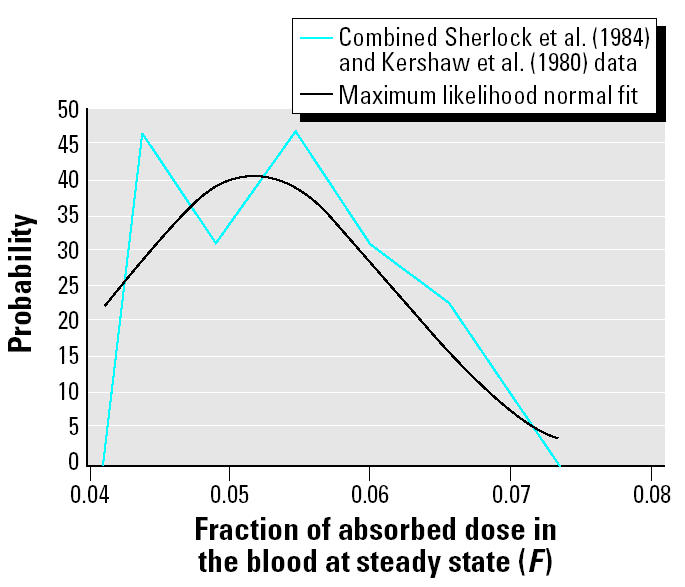
Comparison of combined Sherlock et al. (1984) and Kershaw et al. (1980) data on fraction of the dose in maternal blood to the maximum likelihood normal distribution.

The combined Sherlock et al. (1984) and Kershaw et al. (1980) data are consistent with both normal and log-normal distributions by standard goodness-of-fit tests. There is little difference in how well these data are fitted by these distributions. This may be a function of the relatively small size of the combined data set (*n* = 24). In the absence of further indications, the normal distribution is chosen to avoid extreme values in the more elongated upper tail of the log-normal distribution. Figure 4 shows the fit of the maximum likelihood normal distribution to these data.

Given the lack of pregnancy-specific data for this parameter, the multiple uncertainties in deriving the parameter from the available data, the incompatibility of one of the three available data sets, and the uncertainty in the specification of the distributional form, this parameter is assigned a high true uncertainty estimate.

### W—*Maternal body weight.*

Although cord blood Hg concentration largely reflects maternal intake during the third trimester, the changing nature of maternal body weight during this period and the lack of a unique reference point during this period, other than delivery, make maternal weight at delivery the most appropriate measure of maternal weight for this analysis. Directly measured population-based data on maternal weight at delivery for the U.S. population are not available. The Centers for Disease Control and Prevention (CDC) does, however, collect individual-specific data from 19 participating states on weight gain during pregnancy and prepregnancy weight as part of their Pregnancy Risk Assessment Monitoring System (PRAMS) database (CDC 2004b). Data for both parameters are partly self-reported. Maternal weight at delivery is calculated as the sum of these two quantities. Although the databases for weight gain and prepregnancy weight are intrinsically linked, corresponding data for individuals were available only through internal CDC codes. Linked data were therefore provided directly in tabulated form (Whitehead N, personal communication). These data are given in Table 3.

Although these data are specific to delivery, the fact that they result partly from self-reported information produces some uncertainty. In addition, they represent data from only 19 of the 50 U.S. states. There is also a suggestion of some uncertainty in the tails of the distribution. Overall, this parameter is associated with a medium degree of true uncertainty.

## Results

### Model Outputs

Table 4 summarizes the distribution of the values estimated for *D*, the maternal intake dose corresponding to 58 μg Hg/L cord blood. Values are the mean of five separate simulations. The last two rows in Table 4 express the variability in the estimate of the maternal dose relative to its central tendency estimate. The ratio of the 50th percentile estimate to the 5th percentile estimate reflects the “distance” between the median maternal dose and the maternal dose at which 95% of the fetal population is predicted to achieve 58 μg/L (i.e., the lower 5% estimate of dose). Likewise, the ratio of the 50th percentile to the 1st percentile reflects the distance between the median maternal dose and the maternal dose at which 99% of the fetal population is predicted to achieve 58 μg/L (i.e., the lower 1% estimate of dose). Based on this analysis, the lower 5% estimate of dose is about a factor of 3 lower than the median dose, and the lower 1% estimate of dose is a factor of 4 lower than median dose.

### Sensitivity Analyses

The derivation of the distributions for each of the input parameters is associated with varying degrees of true uncertainty (i.e., lack of knowledge). In the context of this analysis, this includes both the uncertainty in estimating the true values of the central tendency and percentiles of the input parameters, and to the uncertainty in specifying their correct distributional form. In theory, true uncertainty is reducible through the use of more or better data. On the other hand, variability is an inherent property of the data and is not reducible through more or better data.

#### Sensitivity analysis of variability.

Table 5 presents a sensitivity analysis of the contributions of each of the model input parameters to the variability in the model output. This is accomplished in the Monte Carlo simulation by setting each model parameter, in turn, to its fixed (point estimate) mean value—that is, by removing each parameter’s contribution to the output variability. The resulting model output is then compared with the model output reflecting the contribution of all of the parameters to the output variability. The magnitude of the percent difference between these outputs is a measure of the contribution of each parameter to the total variability. A negative percent difference indicates that the parameter functions in the full model to increase variability, and a positive difference indicates that the parameter functions to decrease variability. In this analysis, differences in the outputs are assessed using the ratio of the 50th percentile value to the 5th or 1st percentile value (i.e., the normalized lower 95th, and 99th percentiles of the maternal intake dose resulting in 58 μg/L Hg in cord blood). This reflects the variability in the portion of the output containing the sensitive population. Clearly, *R* makes the largest contribution to output variability, with much smaller contributions (in decreasing order) by *b*, *F*, and *W*. *V* and *A* make no significant contribution to the variability.

Table 5 also presents a summary of the estimates of true uncertainty for each of the input parameters as discussed in the preceding section. Considering the contributions of each parameter to output variability and true uncertainty together, *R*, the cord blood:maternal blood Hg ratio, is the parameter that clearly has the greatest influence on variability. However, it is associated with a low level of true uncertainty and thus makes little contribution to uncertainty in output variability. The elimination rate constant, *b*, makes the next largest contribution to variability, but its influence is only about 20% of *R*. It is associated with a moderate level of true uncertainty. Its overall contribution to uncertainty in the output variability is therefore relatively small. *F*, the fraction of the dose residing in the blood, is highly uncertain but makes only a small contribution to variability. Its contribution to uncertainty in output variability is therefore also relatively small. All other parameters add little or no uncertainty to the output variability.

#### Sensitivity analysis of central tendency.

If the central tendency estimates of the input parameters (i.e., means, medians) in this analysis are inaccurate, they could bias the estimates of the central tendency and percentiles of the model output even if the variability in the output distribution is accurately estimated. Because three of the input parameters are judged to have a low degree of true uncertainty (Table 5), it is unlikely that they will contribute significantly to uncertainty in the central tendency estimate of the output. Therefore, I focused on the variables with medium or high uncertainty. Because *b* and *W* are judged to have medium uncertainty, I assumed, for the purposes of this analysis, that the true mean value could vary from their original estimate by ± 10%. For *F*, with high uncertainty, it was assumed that the true mean value could vary by ± 20% from its original estimate. These parameters were therefore allowed to assume one of three fixed values: their original estimate of mean value, +10% (or 20% for *F*) of their original estimate, and –10% (or 20% for *F*) of their original estimate. The remaining parameters were fixed at the original estimates of their mean values. The model was calculated using Monte Carlo sampling (5,000 iterations) to yield the various possible combinations of mean maternal dose. Table 6 presents the results of this analysis.

The minimum and maximum changes reflect the outcomes where the central tendencies of each of the input parameters are simultaneously altered in the direction that produces the greatest change in the output. The values between the 25th and 75th percentiles reflect a more likely combination of altered input values resulting in a more likely estimate of the uncertainties in the central tendency estimates. Under the assumptions of this analysis, the true uncertainty in the central tendency estimates of the most uncertain input parameters is most likely to influence the estimate of maternal dose by ≤ 20%. In addition, because of the unexplained difference in the value of *F* estimated from the Smith et al. (1994) data compared with the combined Kershaw et al. (1980) and Sherlock et al. (1984) data, this analysis was rerun assuming that the maximum value of *F* could be 48% larger than the original central tendency estimate [i.e., equal to the mean value from the analysis in Smith et al. (1994)]. The results of this analysis (not shown) indicate that, as expected, the use of a larger value of *F* results in a smaller value at the lower end of the range of likely outcomes. The difference, however (a 28% decrease at the 25th percentile compared with the 20% decrease seen for the analysis presented in Table 6), is not dramatic and suggests that, in the context of the overall uncertainty in the estimate of central tendency, incorporating the Smith et al. (1994) value for *F* would have a relatively small effect on the central tendency estimate.

## Discussion

This reanalysis of the maternal MeHg dose resulting in 58 μg Hg/L in fetal cord blood was undertaken to address two specific needs that have arisen since the original NRC and U.S. EPA analyses. The first was to incorporate the cord blood:maternal blood ratio in the analysis. This was accomplished by integrating the distribution of *R* derived in the recent analysis of Stern and Smith (2003). The second need was to reduce the uncertainty in the estimate of the central tendency of the maternal dose. This was accomplished by reassessing each of the input parameters in the one-compartment model. This also allowed a reassessment of the variability in the estimate of maternal dose. As expected based on both its central tendency estimate of 1.7 (as opposed to the original implicit estimate of 1.0) and the significant variability around this value (Stern and Smith 2003), the cord blood:maternal blood Hg ratio (*R*) had a significant influence on the estimate of maternal dose. In addition to proportionally decreasing the central tendency estimate, this parameter significantly increased the variability in the estimate, resulting in a 37% increase in “distance” between the median and the lower 5th percentile and a 49% increase in the distance between the median and the lower 1st percentile. The sensitivity analysis of variability shows that this parameter is the largest source of variability in the estimate of maternal dose.

Much of the previous uncertainty in the central tendency estimate of maternal dose resulted from the lack of temporal specificity in the model parameters regarding the period of pregnancy. In the present analysis, I made a specific effort to identify distributional data that are specific to pregnancy and, whenever possible, specific to the third trimester of pregnancy—the period of gestation corresponding most closely to the period of accumulation of measured Hg in cord blood. Appropriate pregnancy-specific data were identified for four of the six parameters in the model (excluding *C*, the empirical value selected as the point of departure). Third-trimester–specific data sets were identified for three of these four. As shown in Table 7, the two largest differences in central tendency values between the present analysis and the U.S. EPA analysis are for *R* and *W*. The value of *W* used in the present analysis is 21% larger than the value chosen by the U.S. EPA. The value used in the present analysis is based on recent data that are specific to delivery and are reasonably consistent with independent estimates of pregnancy weight gain and prepregnancy weight. *R* is entered into the numerator of the model as its reciprocal, and *W* appears in the denominator of the model. Thus, the effect of increasing the value of both of these parameters relative to the U.S. EPA estimates is to decrease the central estimate of the maternal dose. Based on the values in Table 7, the central tendency estimate of maternal dose corresponding to 58 μg Hg/L cord blood is 0.7 μg/kg/day. Note that this value differs from the mean value derived from the full distributional analysis (Table 4), because the equation for the model is nonlinear, and therefore, the mean of the means (i.e., the central tendency estimate) is not equivalent to the overall mean. The corresponding U.S. EPA central tendency estimate is 1.1 μg/kg/day (U.S. EPA 2001). When both *R* and *W* are set to their U.S. EPA central tendency values, the effect of the remaining central tendency values from the present analysis is to increase the dose estimate to 1.4 μg/kg/day. Thus, although individually the changes in *b*, *V*, *F*, and *A* are relatively small compared with their values in the U.S. EPA analysis, the combined effect of the changes is influential. Apart from the extent of change in the central tendency estimates, the increased specificity and in-depth evaluation presented here can reduce the level of uncertainty present in the existing U.S. EPA analysis.

In addition to the reassessment of the central tendency estimates, this analysis yields a revised analysis of the variability in the estimate of maternal dose. The analysis presented as part of the NRC report (2000) and discussed in Stern et al. (2002) concluded that dividing the central tendency estimate of dose by a factor of 2–3 would account for 95–99% of the variability in the dose estimate. Based largely on that analysis, the U.S. EPA chose a value for the 50th percentile:1st percentile ratio of maternal dose of 3.0 (U.S. EPA 2001). The central tendency estimate was divided by this value to obtain an estimate of the 1st percentile (i.e., lowest 99th percentile) of maternal intake dose. In the present analysis, a value of 4.0 is estimated for the 50th percentile:1st percentile ratio, and a value of 2.7 for the 50th percentile:5th percentile ratio (Table 4).

To address the uncertainties in the estimates of the central tendency of the individual model parameters, the U.S. EPA followed the recommendation of the NRC to decouple the central tendency and variability estimates of maternal dose. By doing so, the U.S. EPA could explicitly discuss central tendency values and separately fold the variability into the overall consideration of uncertainty factors. For a cord blood Hg concentration of 58 μg/L, this approach yields an estimate of the 1st percentile of maternal dose of 0.4 μg/kg/day (i.e., 1.1 μg/kg/day/3.0) based on the U.S. EPA’s central tendency and variability estimates. To the extent that the present analysis has substantially reduced the uncertainty in the estimates of central tendency, it can be argued that this decoupling is no longer necessary. Rather, I believe that it is now appropriate to directly estimate the lowest 1st (or any other) percentile of maternal dose corresponding to 58 μg Hg/L cord blood directly from the output of the one-compartment model. With reference to Table 4, a maternal intake dose of 0.2 μg/kg/day would correspond to the 1st percentile, and a dose of 0.3 μg/kg/day would correspond to the 5th percentile. Thus, the present analysis supports an estimate that is half of the value implicit in the U.S. EPA analysis. Following this approach, no uncertainty factor would be required to account for pharmacokinetic variability in the estimate of maternal dose in the calculation of the RfD. Other uncertainty factors could still be applied to the derivation of the RfD as appropriate to address database factors as well as the remaining fetal variability in the system including toxicodynamic factors.

## Figures and Tables

**Figure 5:**
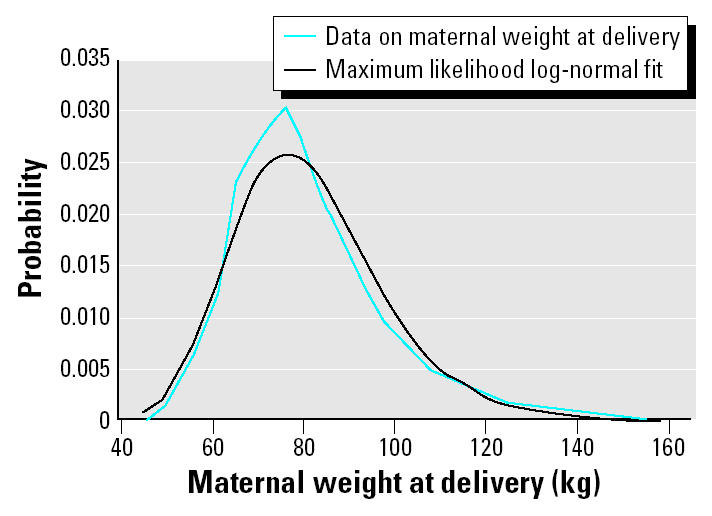
Comparison of maternal delivery weight data (Whitehead N, personal communication) to the maximum likelihood log-normal distribution.

**Table 1:** Summary of distributions selected for parameters of the one-compartment model.

		Distributions	
Parameter	Source	Data	Probability
*C* (concentration of Hg in cord blood)	NRC 2000	58 μg/L	
*R* (ratio of Hg concentration in cord blood to maternal blood	Stern and Smith 2003		Log-normal
			Mean ± SD = 1.7 ± 0.9
*b* [rate constant for elimination of MeHg from blood; note that this parameter is reported as half-life of MeHg in blood (*T*_1/2_), related to *b* as *b* = ln 0.5/*T*_1/2_]	Cox et al. 1989	Empirical *T*_1/2_ (days)	Relative probability
		20	2.46
		25	1.64
		30	5.74
		35	8.20
		40	12.30
		45	17.21
		50	14.75
		55	25.41
		60	7.38
		65	4.10
		70	0.82
		Minimum = 15	
		Maximum = 75	
*V* (maternal blood volume)	Thomson et al. 1938, Caton et al. 1951	Empirical (correlated with *W*) *V* (L)	Cumulative probability
		4.480	0.05
		4.530	0.1
		4.970	0.25
		5.280	0.5
		6.310	0.75
		6.408	0.85
		6.694	0.9
		7.380	0.95
		Minimum = 3.707	
		Maximum = 7.902	
		Correlated with *W* – *r* = 049	
*A* (fraction of dose absorbed)	Miettinen et al. 1971 (modified to account for metabolism)	Empirical *A*	Cumulative probability
		0.947	0.071
		0.960	0.286
		0.971	0.5
		0.983	0.786
		0.996	0.929
		Minimum = 0.940	
		Maximum = 0.999	
*F* (fraction of absorbed dose that is present in the blood at steady state)	Sherlock et al. 1984, Kershaw et al. 1980		Normal
			Mean ± SD = 0.052 ± 0.0095
*W* (maternal body weight)	CDC 2004b		Log-normal
			Mean ± SD = 80.9 ± 16.3 kg
			Correlation with *V* – *r* = 0.49

**Table 2:** MeHg half-life and elimination rate as a function of intake dose.

Study	No.	Mean dose μg/kg/day (range)	Mean *T*_1/2_ (days)	Mean elimination rate constant (*b*)
Cox et al. 1989	55	(1.2–79.6)*^a^*	47.2	0.0147
Miettinen et al. 1971	6	0.3*^b^*	49.9	0.0142
Sherlock et al. 1984	20	1.6 (0.5–3.6)	50.2	0.0140
Kershaw et al. 1980	4	20.0 (18.1–20.9)	53.0	0.0133

a Estimated from reported hair Hg concentrations based on the one-compartment pharmacokinetic model, and assuming a 62-kg body weight.

b Estimated assuming uniform intake of the tracer dose and 70-kg body weight.

**Table 3:** Calculated percentiles of maternal weight (kg) at delivery (CDC 2004b; Whitehead N, personal communication).

Cumulative percentile	Maternal weight at delivery (based on PRAMS data)
1st	52.66
5th	59.02
10th	63.00
20th	67.36
30th	71.11
40th	74.59
50th	77.91
60th	81.62
70th	86.23
80th	92.07
90th	102.15
95th	112.64
99th	135.93

**Table 4:** Model output of selected percentiles of the maternal intake dose of MeHg corresponding to 58 μg Hg/L cord blood.

Distribution of maternal MeHg intake dose (percentile)	Maternal intake dose (μg/kg/day)
Mean ± SD	0.993 ± 0.702
1st	0.202
5th	0.301
10th	0.373
50th	0.812
50th/5th	2.700
50th/1st	4.020

**Table 5:** Estimated contributions of model parameters to variability*^a^* and true uncertainty*^b^* in the model output.

Contribution	*R*	*b*	*V*	*A*	*F*	*W*
50th percentile/5th percentile	−37.0	−8.3	< 0.1	+0.4	−5.9	−2.0
50th percentile/1st percentile	−49.1	−9.8	+0.3	−1.4	−7.2	−2.1
True uncertainty	L	M	L	L	H	M

See text (“The One-Compartment Model”) for an explanation of the model paremeters.

a Percent change in normalized 5th and 1st percentile values for models with fixed parameters compared to the full variability model.

b Estimates of the degree of true uncertainty in the specification of the model parameters: H, high; M, medium; L, low.

**Table 6:** Sensitivity analysis of the estimates of the central tendency for selected model parameters.

Outcomes from the combination of alternative central tendency estimates for selected model parameters	Estimated mean dose	Percent change from original mean dose
Original mean maternal dose	0.71*^a^*	—
Minimum alternative value	0.47	−34
Maximum alternative value	1.04	+46
25th percentile alternative value	0.57	−20
75th percentile alternative value	0.84	+18

Values represent the change in the mean maternal dose resulting from various combinations of high and low values for input parameters. See text (“Sensitivity analysis of central tendency”) for explanation of parameter selection.

a This value differs from the mean estimated from the full distributional analysis.

**Table 7:** Comparison of central tendency estimates in the present analysis to central tendency estimates in the U.S. EPA RfD derivation (U.S. EPA 2001).

Parameter	Current central*^a^* tendency estimate	Pregnancy specific?	Third-trimester specific?	EPA central tendency estimate	Pregnancy specific?	Third-trimester specific?
*R*	1.7	Yes	Yes	1.0 (implicit)	No	No
*b*	0.0147 day^−1^ (47 days)	Yes	No	0.014 day^−1^ (50 days)	No	No
*V*	5.6 L*^b^*	Yes	Yes	5 L*^c^*	Yes	Yes
*W*	80.9 kg	Yes	Yes	67 kg	Yes	No
*A*	0.97	No	No	0.95	No	No
*F*	0.052	No	No	0.059	No	No

a Means of fitted distributions (see Table 1).

b U.S. women.

c Nigerian women.
